# Huntingtin-associated protein 1: Eutherian adaptation from a TRAK-like protein, conserved gene promoter elements, and localization in the human intestine

**DOI:** 10.1186/s12862-016-0780-3

**Published:** 2016-10-13

**Authors:** Amanda L. Lumsden, Richard L. Young, Nektaria Pezos, Damien J. Keating

**Affiliations:** 1 Centre for Neuroscience and Department of Human Physiology, Flinders University, Adelaide, South Australia Australia; 2 South Australian Health and Medical Research Institute, Adelaide, South Australia Australia; 3 Department of Medicine, University of Adelaide, Adelaide, South Australia Australia

**Keywords:** HAP1, TRAK1, TRAK2, Milton, CDX1, CDX2, TATA-binding protein, Myc-associated factor X, Serotonin, Enterochromaffin

## Abstract

**Background:**

Huntingtin-associated Protein 1 (HAP1) is expressed in neurons and endocrine cells, and is critical for postnatal survival in mice. HAP1 shares a conserved “HAP1_N” domain with TRAfficking Kinesin proteins TRAK1 and TRAK2 (vertebrate), Milton (*Drosophila*) and T27A3.1 (*C. elegans*). HAP1, TRAK1 and TRAK2 have a degree of common function, particularly regarding intracellular receptor trafficking. However, TRAK1, TRAK2 and Milton (which have a “Milt/TRAK” domain that is absent in human and rodent HAP1) differ in function to HAP1 in that they are mitochondrial transport proteins, while HAP1 has emerging roles in starvation response. We have investigated HAP1 function by examining its evolution, and upstream gene promoter sequences. We performed phylogenetic analyses of the HAP1_N domain family of proteins, incorporating HAP1 orthologues (identified by genomic synteny) from 5 vertebrate classes, and also searched the *Dictyostelium* proteome for a common ancestor. Computational analyses of mammalian HAP1 gene promoters were performed to identify phylogenetically conserved regulatory motifs.

**Results:**

We found that as recently as marsupials, HAP1 contained a Milt/TRAK domain and was more similar to TRAK1 and TRAK2 than to eutherian HAP1. The Milt/TRAK domain likely arose post multicellularity, as it was absent in the *Dictyostelium* proteome. It was lost from HAP1 in the eutherian lineage, and also from T27A3.1 in *C. elegans*. The HAP1 promoter from human, mouse, rat, rabbit, horse, dog, Tasmanian devil and opossum contained common sites for transcription factors involved in cell cycle, growth, differentiation, and stress response. A conserved arrangement of regulatory elements was identified, including sites for caudal-related homeobox transcription factors (CDX1 and CDX2), and myc-associated factor X (MAX) in the region of the TATA box. CDX1 and CDX2 are intestine-enriched factors, prompting investigation of HAP1 protein expression in the human duodenum. HAP1 was localized to singly dispersed mucosal cells, including a subset of serotonin-positive enterochromaffin cells.

**Conclusion:**

We have identified eutherian HAP1 as an evolutionarily recent adaptation of a vertebrate TRAK protein-like ancestor, and found conserved CDX1/CDX2 and MAX transcription factor binding sites near the TATA box in mammalian HAP1 gene promoters. We also demonstrated that HAP1 is expressed in endocrine cells of the human gut.

**Electronic supplementary material:**

The online version of this article (doi:10.1186/s12862-016-0780-3) contains supplementary material, which is available to authorized users.

## Background

Huntingtin-associated Protein 1 (HAP1) is a ~100 kDa protein that was first identified in a screen for proteins that interact with the Huntington’s disease (HD) gene product, huntingtin (HTT) [1]. Initial interest in HAP1 stemmed from its potential role in HD pathogenesis, and has broadened due to its emerging roles in feeding and metabolism [2], and association with a range of conditions (in addition to HD) including depression [3], autism [4], Alzheimer’s disease [5], Joubert syndrome [6], Rett Syndrome [7], and cancer [8]. The biological function of HAP1 remains to be fully elucidated, and is of potential importance to a number of research fields given the wide-ranging disease associations of this protein.

HAP1 is expressed primarily in neurons [9], and in endocrine cells of the periphery [10, 11]. HAP1 is also diffusely expressed in the mucosal layer of the stomach and small intestine in as-yet unidentified cells [10]. At the intracellular level, HAP1 is associated with organelle transport [12–14], promotes neurite outgrowth [15], is transported along nerve axons [16] and facilitates receptor internalization [6, 15, 17]. HAP1 achieves this through interactions with binding partners involved in intracellular transport, such as kinesin light chain (KLC), dynactin subunit p150^glued^ [15], dynein [18] and synapsin I [19]. Through linking to cytoskeletal motor proteins, HAP1 acts in the trafficking of various cargoes such as GluR2-containing AMPA receptors [20], EGF receptors [21], GABA_A_ receptors [22], BDNF [23], proBDNF [14], amyloid precursor protein [5] and β-actin RNA [18], and also plays a role in ciliogenesis [24]. Such cytoskeletal motor interactions likely explain how HAP1 promotes the release of hormones such as insulin [25], and catecholamines [26], as well as neurotransmitters [19], through the regulation of vesicle transport to the plasma membrane.

Recent studies support a role for HAP1 in the promotion of energy conservation. HAP1 stabilizes levels of an inhibitor of mTORC1 [4], suppression of which is associated with the activation of autophagy. HAP1 localizes to autophagosomes to promote their trafficking along axons [12]. *Hap1* gene expression increases in response to fasting [2], and conversely, insulin mediates HAP1 degradation in orexigenic neurons of the hypothalamus [2], suggesting that the demand for HAP1 is reduced post feeding. These findings are consistent with a role for HAP1 in response to caloric insufficiency.

Newborn *Hap1* ^*−/−*^ mouse pups fail to ingest milk, leading to postnatal starvation and death [27–29]. Conditional *Hap1* gene inactivation in hypothalamic orexin neurons decreases food consumption and body weight [30], and hypothalamic neurodegeneration and decreased neurogenesis occur in mouse pups deficient in HAP1 [17, 28]. However, no defect in the hypothalamic expression of genes encoding appetite-regulating neuropeptides (AGRP, POMC and NPY) has been detected in *Hap1* ^*−/−*^ mice [29] and as yet, the mechanism by which HAP1 deficiency causes postnatal failure to feed and thrive has not been established.

HAP1 is one of a group of related proteins also including vertebrate TRAfficking Kinesin-binding proteins TRAK1 (previously known as O-linked N-acetylglucosamine transferase interacting protein 106, OIP106) and TRAK2 (previously Gamma-aminobutyric acid(A) receptor-interacting factor, GRIF-1), the *Drosophila* Milton protein, and T27A3.1 in *C. elegans*. These proteins share a homologous region [31, 32] termed the HAP1_N domain (Pfam ID: PF04849). TRAK1, TRAK2 and Milton, however, contain an additional homologous region on the carboxyl side of the HAP1_N domain called the Milton/Trafficking kinesin-associated protein domain (Milt/TRAK domain; Pfam ID: PF12448), a domain that is absent in human and rodent HAP1 proteins. The HAP1_N domain is rich in coiled-coil motifs and facilitates the interaction of HAP1 with the coiled-coil domains possessed by many HAP1-interacting proteins including HTT [1, 33, 34], p150^glued^, PCM1 [35], Hrs [21], Kinesin-1 family member KIF5 [36], kinesin light chain [37], and HAP1 itself [38]. Vertebrate TRAK proteins and *Drosophila* Milton interact with some of the same proteins/protein families as HAP1, and have overlapping functions. For example TRAK1 interacts with Hrs and is involved in EGFR sorting [39]. TRAK1 and TRAK2 interact with GABA_A_ receptors and are implicated in their trafficking [40, 41]. TRAK1 and 2, and Milton bind to kinesin-1 family proteins *via* the HAP1_N-containing N-terminal region [32, 42, 43] and interact with dynein/dynactin proteins [44] implicating them in anterograde and retrograde transport. Like HAP1, TRAK1 and TRAK2 proteins are involved in neuritic outgrowth (of axons and dendrites, respectively) [44] and are also able to self-interact [45].

In contrast to HAP1 however, TRAK1, TRAK2 and *Drosophila* Milton have a prominent role in mitochondrial trafficking. They link mitochondria to kinesin motor proteins *via* a complex with the Rho-like GTPase Miro, to facilitate transport of the mitochondria to nerve terminals, axons and dendrites to provide targeted provision of energy to localized areas within the cell [32, 43, 44, 46]. Although immunogold electron microscopy has shown HAP1 localizes to mitochondria [13], HAP1 has not been associated with mitochondrial targeting to neurites. In contrast to HAP1, the Milt/TRAK proteins have not been linked to starvation response and feeding.

In this study we investigated HAP1 function by assessing the evolution and divergence of HAP1_N protein family members, and conserved regulatory elements in mammalian HAP1 gene promoters. The online access to increasing numbers of sequenced and annotated genomes allowed inclusion of orthologues (determined by genomic synteny) representing the 5 vertebrate classes. Furthermore, since little is known about regulation of HAP1 gene expression, we also searched eight mammalian HAP1 gene upstream promoter sequences for regulatory elements and multi-element configurations (promoter models), using phylogenetic conservation to provide evidence for functionality. Finally, upon detecting a highly conserved promoter model near the start of transcription containing sites for intestine-enriched transcription factors, we examined HAP1 protein expression in the human duodenum.

## Results

### Verification of *HAP1* orthologous genes in vertebrates

**Fig. 1:**
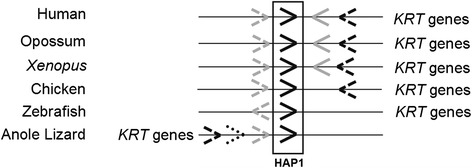
Schematic map of *HAP1* chromosomal regions. Genes are depicted as arrows in direction of gene orientation. *HAP1* (*black*), *JUP* (*gray dashed*), *GAST* (*gray solid*), *EIF1* (*black dashed*) *LEPREL4* (*black dotted*) genes are shown. The genome sequences are from human (*H. sapiens, chr17*), opossum (*M. domestica, chr2*), clawed frog (*X. tropicalis, chrUnknown,* contig NW_004668245.1), chicken (*G. gallus, chr27*), zebrafish (*D. rerio, chr11*) and anole lizard (*A. carolinensis, chr6).* Not to scale

Searches for *HAP1* orthologues were performed of the genomes of the following species: human (*Homo sapiens*), rat (*Rattus norvegicus*), mouse (*Mus musculus*), dog (*Canis familiaris*), horse (*Equus caballus*), rabbit (*Oryctolagus cuniculus*), opossum (*Monodelphis domestica*), Tasmanian devil (*Sarcophilus harrisii*), zebrafish (*Danio rerio*), chicken (*Gallus gallus*), anole lizard (*Anolis carolinensis*) and clawed frog (*Xenopus tropicalis*). From here on, these species are referred to by their common names, except for *Xenopus*. These genome representatives of the 5 classes of vertebrate, including eutherian and marsupial mammals, were examined using the Ensembl genome browser [47] and were each found to contain three HAP1_N domain-containing genes. These genes were *TRAK1*, *TRAK2*, and a third gene that at the outset of this study was named *HAP1* or *TRAK1-like*, or was unannotated, depending upon the species. In order to confirm whether the *TRAK1-like* and unannotated genes were orthologous to human *HAP1*, genomic contexts were compared for verification (Fig. 1, selected species shown). In human, the *HAP1* gene was found to be flanked by the genes *JUP* (upstream, on *HAP1* sense strand) encoding Junction Plakoglobin, and *GAST* (encoding Gastrin, downstream, on antisense strand) followed by *EIF1* further downstream (encoding Elongation Initiation Factor 1, antisense strand), and a cluster of cytokeratin (*KRT*) genes (sense strand; Fig. 1). This genomic context was distinct from those of *TRAK1* and *TRAK2* (not shown). The same gene context was observed for *Hap1* genes of other eutherian mammals (rat, mouse, dog, horse, rabbit), marsupials (opossum *Trak-1 like protein* gene and Tasmanian devil *Hap1*) and amphibian (*Xenopus hap1*). A similar arrangement was also observed in bird (chicken unannotated gene, ENSGALG00000023847) except that the *Gast* gene was absent. In fish (zebrafish), the HAP1_N- containing unannotated gene (ENSDARG00000074508) was positioned between *jupa* (upstream) and *krt* genes (downstream) consistent with other *HAP1* orthologous genes, and was therefore considered a homolog. The *gast* and *eif1* genes were absent in the region in zebrafish, and *jupa* was on the antisense strand, in contrast to other vertebrates. In reptile (anole lizard), *jup* (sense) was upstream of *hap1* (with an uncharacterized locus between them) and *eif1* (sense) was further upstream of *jup*, with additional genes (*leprel4*, and 3 non-coding RNA sequences) between them. In the lizard the cluster of *krt* genes was upstream of *eif1* as in human, opossum, and *Xenopus*, but the *krt* cluster and *eif* gene were upstream rather than downstream of *HAP1* (Fig. 1). No *gast* gene was present in the anole lizard. In summary, all vertebrates investigated contained three HAP1_N genes *TRAK1*, *TRAK2* and a third gene in a comparable genomic context to human *HAP1*. In a more recent version of the Ensembl browser (Version 83, December 2015), the TRAK1-like and ‘unannotated’ orthologues described above have been renamed HAP1*,* consistent with our findings.

### Non-eutherian vertebrate HAP1 proteins contain a TRAK/Milton domain

**Fig. 2:**
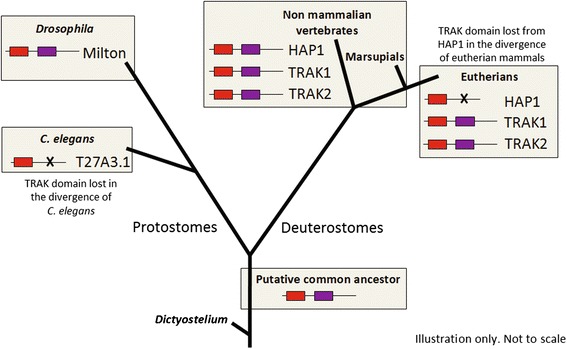
Evolutionary conservation of HAP1_N and Milt/TRAK domains. A common ancestor to TRAK-like proteins is predicted to exist prior to the divergence of protostomes and deuterostomes containing a HAP1_N domain (*red*) and Milt/TRAK domain (*purple*). In protostomes, the Milt/TRAK domain has been lost from the single homolog in *C. elegans* (T27A3.1), but has been maintained in *Drosophila* Milton. In vertebrates, gene duplication has given rise to 3 paralogous genes encoding HAP1, TRAK1 and TRAK2, prior to the divergence of the 5 classes of vertebrate. In non-eutherian vertebrates, HAP1 is a TRAK-like protein with a HAP1_N and Milt/TRAK domain. The TRAK domain has subsequently been lost in the divergence of eutherian mammals

The presence of predicted domains within the amino acid sequence of vertebrate HAP1 proteins was compared using Ensembl [47] and NCBI’s conserved domain database [48]. Whilst all vertebrate HAP1 proteins contained the characteristic HAP1_N domain, HAP1 proteins from marsupial (opossum and Tasmanian devil) and non-mammalian vertebrates (*Xenopus*, zebrafish, chicken and anole lizard) also contained a Milt/TRAK domain characteristic of TRAK1, TRAK2 and *Drosophila* Milton. HAP1 proteins from human and other eutherian mammals (rat, mouse, rabbit, dog and horse) all lacked the Milt/TRAK domain. Interestingly, the Milt/TRAK domain was also absent in T27A3.1, suggesting the domain was lost a second time, in the divergence of *C. elegans* (Fig. 2).

### Sequence comparisons of HAP1_N protein family members

Sequence comparisons were performed using HAP1 proteins from selected eutherian mammals (human, dog, rat), marsupials (opossum, Tasmanian devil), and non-mammalian vertebrates (chicken, anole lizard, *Xenopus*, zebrafish), TRAK1 and TRAK2 proteins from human and opossum, and the *C. elegans* and *Drosophila* homologs. The complete identity matrix is shown in Additional file 1. Human HAP1 showed most similarity overall to HAP1 from dog (67 %) and rat (65 %). Opossum HAP1, which was highly similar to Tasmanian devil HAP1 (84 %), was more similar to HAP1 proteins from non-mammalian vertebrates (48–58 % identity) than to human HAP1 (39 %). In contrast, high homology was observed between opossum and human TRAK1 (92 %) and TRAK2 (83 %) proteins. In opossum, HAP1 was more similar to its paralogs TRAK1 (46 %) and TRAK2 (39 %) than human HAP1 was to its paralogs (29 and 26 %, respectively). HAP1 proteins from non-human eutherian mammals (rat and dog) were more closely related to human HAP1 (65, 67 %) than to opossum HAP1 (37, 37 %), as expected by their lack of Milt/TRAK domain. These results were consistent with a eutherian adaptation of HAP1 sequence since divergence from marsupials. HAP1 proteins from eutherian mammals (human, rat, dog), lacking the Milt/TRAK domain, were shorter in length (599–679aa) than vertebrate HAP1 orthologues that contained the Milt/TRAK domain (opossum, Tasmanian devil, chicken, lizard, *Xenopus* and zebrafish; 708–901aa). The latter were closer in length to vertebrate TRAK1 (860–1036aa) and TRAK2 (884–993aa) proteins. An alignment of all protein sequences in this analysis is shown in Additional file 2, indicating the position of the HAP1_N and Milt/TRAK domains.

**Fig. 3:**
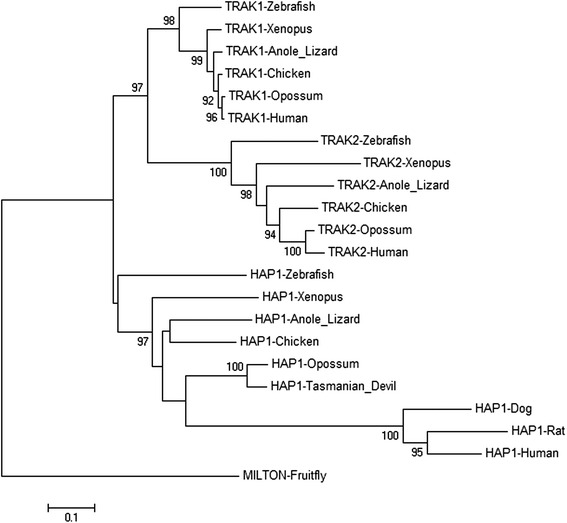
Phylogenetic relationship of HAP1_N domains of selected vertebrates and *Drosophila* HAP1_N family members. Multiple sequence alignment was performed with the ClustalW program. The phylogenetic tree was constructed using the neighbor-joining method. The tree was rooted using the *Drosophila* Milton HAP1_N domain sequence as an outgroup. The percentage of replicate trees in which the associated taxa clustered together in the bootstrap test (1000 replicates) is shown next to the branches, where equal to or greater than 90 %. Sequence distance is indicated by horizontal arm length, scale bar shown. The HAP1_N domains from the following sequences were used: human (*H. sapiens* HAP1: ENSP00000334002.4 aa108–428; TRAK1: ENSP00000328998.5 aa48–354; TRAK2: ENSP00000328875.3 48–353), Tasmanian devil (*S. harrisii* HAP1: XP_012403736.1 aa9–292), rat (*R. norvegicus* HAP1: ENSRNOP00000072494.1 aa81–403), dog (*C. familiaris* HAP1: ENSCAFP00000023416.4 aa100–465), opossum (*M. domesticus* HAP1: XP_007482271.1 aa9–292; TRAK1: ENSMODP00000013909.4 aa47–352; TRAK2: ENSMODP00000018999.3 aa48–353), zebrafish (*D. rerio* HAP1: ENSDARP00000099219.3 aa19–303; TRAK1: ENSDARP00000115803.1 aa1–250; TRAK2: ENSDARP00000133065.1 aa32–349), clawed frog (*Xenopus tropicalis* HAP1: ENSXETP00000027307.2 aa116–422; TRAK1: XP_012819699.1 aa36–296; TRAK2: ENSXETP00000061179.1 aa29–335), chicken (*G. gallus* HAP1: ENSGALP00000039222.2 aa122–425; TRAK1: ENSGALP00000019447.4 aa129–435; TRAK2: ENSGALP00000013618.4 aa30–333), anole lizard (*A. carolinensis* HAP1: XP_008111605.1 aa10–305; TRAK1: XP_008113765.1 aa33–254; TRAK2: ENSACAP00000001540.3 aa30–353), fruitfly (*Drosophila melanogaster* Milton: FBpp0297338.1 aa76–375)

A phylogram was compiled of isolated HAP1_N domain sequences from TRAK1, TRAK2 and HAP1 proteins from human, opossum, *Xenopus*, zebrafish, chicken and anole lizard, as well as dog and rat (HAP1 only). The HAP1_N domain of Milton was included as a more distant reference sequence for rooting the tree (Fig. 3). HAP1_N sequences from vertebrate TRAK1 orthologues clustered closely together in a single clade, as did those from vertebrate TRAK2 proteins in another clade. In contrast, the HAP1_N domain sequences of vertebrate HAP1 proteins were more divergent. The HAP1_N domain from eutherians had changed more substantially than that of marsupials since their last inferred common ancestor, as illustrated by an approximate 3-fold increase in phylogram arm length (Fig. 3), suggesting that loss of the TRAK domain altered the selective pressure on the HAP1_N domain.

### Search for a common ancestral protein containing HAP1_N and Milt/TRAK domains prior to divergence of protostomes and deuterostomes

Genes encoding *Drosophila* Milton, and *C. elegans* T27A3.1 in invertebrates, and TRAK1, TRAK2 and HAP1 in vertebrates, are likely to have arisen from a common ancestral gene existing prior to the divergence of protostomes and deuterostomes (Fig. 2). We therefore searched for a common ancestor in the proteome of the early eukaryote *Dictyostelium discoideum* [49]*,* an organism that exists as single cells and as a multicellular entity under different conditions. Searches were performed using as query sequences *Drosophila* Milton and human TRAK1 (the human HAP1_N protein with the highest similarity to Milton). A single likely ancestor was not apparent, however a number of *Dictyostelium* proteins showed significant similarity to the query sequences across the coiled-coil rich HAP1_N domain. No matches were found to other regions of the TRAK1 and Milton query proteins including the Milt/TRAK domain. The majority of the *Dictyostelium* proteins identified were actin-binding proteins (fimbrin and myosin proteins) or kinesin family members, whose homologs interact with TRAK1, TRAK2 and/or Milton proteins. This is consistent with the ability of the HAP1_N domain to facilitate interactions with other coiled-coil proteins including mediating their own self-association.

Actin-binding protein fimbrin-3 (*fimC*) was the most significant match for both queries. Other common hits were myosin II heavy chain (*mhcA*), fimbrin-4 (*fimD*), kinesin family member 4 (*kif4*), DDB_G0286355 (predicted component of the meiotic synaptonemal complex) and DDB_G0289761 (unannotated). These, and other proteins with similarity to either TRAK1 or Milton, and their associated functions, are listed in Table 1. These findings suggest that origins of the HAP1_N domain (present in cytoskeletal-based transport proteins of *Dictyostelium*), pre-dated the evolution of the Milt/TRAK domain which therefore arose post multicellularity, but prior to divergence of protostomes and deuterostomes.

**Table 1:** *D. discoideum* gene products with similarity to the HAP1_N domain region of *Drosophila* Milton and human TRAK1 proteins

	Gene symbol (Gene name description)	Gene product	Description	*E* value (<0.1)
Common Hits	*fimC* (FIMbrin)	Fimbrin-3	Actin binding protein with 4 calponin homology domains	TRAK1: 2×10^−6^ Milton: 2×10^−4^
	*mhcA* (Myosin Heavy Chain A)	Myosin II heavy chain	Myosin II heavy chain, component of the actin-based molecular motor (conventional myosin); has actin-activated ATPase activity;	TRAK1:7×10^−3^ Milton: 3×10^−4^
	*DDB_G0290503*	DDB0346607	Hypothetical protein predicted to be involved in meiosis and the synaptonemal complex	TRAK1: 5×10^−5^ Milton: 0.011
	*fimD* (FIMbrin)	Fimbrin-4	Actin binding protein with 4 calponin homology domains	TRAK1: 0.001 Milton: 0.079
	*kif4* (KInesin Family member 4)	Kinesin family member 4	Member of the CENP-E subfamily, predicted to play a role in mitosis	TRAK1: 0.01 Milton: 0.055
	DDB_G0289761	DDB0348198	Not yet annotated	TRAK1: 0.051 Milton: 0.013
Additional TRAK1 hits	*myoJ* (MYOsin)	myosin-5b	Conditional processive motor protein that can move over long distances along F-actin without disassociating	0.001
	*smc1* (Structural Maintenance of Chromosome)	structural maintenance of chromosome protein	Functions in chromosome dynamics	0.021
	*zipA* (ZIPper)	zipper-like domain-containing protein	-	0.022
	*kif9* (KInesin Family member 9)	Kinesin family member 9	Plays a role in connecting the centrosome with the nucleus	0.067
	*kif1* (KInesin Family member 1)	Kinesin-3	Kinesin family member 1; ortholog of *C. elegans* Unc104 and mammalian KIF1A, involved in intracellular transport	0.09
	*DDB_G0287325*	AP2 complex protein Eps15	Similar to mammalian EPS15, a clathrin adaptor that binds to the AP2 alpha subunit in mammalian cells	0.033
	*DDB_G0288073*	Nucleoprotein TPR	-	0.016
	*DDB_G0271334*	C2 calcium/lipid-binding (CaLB) region and dilute domain-containing protein	Contains a dilute domain, found at the carboxyl terminus of non-muscle myosin V	0.061
Additional Milton hits	*rad50* (Similar to RADiation sensitive mutant)	DNA recombination/repair protein	Similar to *S. cerevisiae* RAD50, involved in processing double-strand DNA breaks with Mre11.	0.001
	*sun1* (Sad1p and UNC-84)	SUN domain-containing protein 1	Localizes to the inner and outer nuclear membrane, mediates the attachment between centrosome and nucleus, associates the centromere cluster with the centrosome, and stabilizes the genome during mitosis; contains one transmembrane domain	0.008
	*ndm* (Negative director of macropinocytosis)	macropinocytosis suppressor Ndm	Contains a coiled-coil, C-terminal BAR-like domain	0.014
	*hook*	hook family protein	Similar to *C. elegans* ZYG-12 and mammalian HOOK proteins, which mediate the attachment between the centrosome and the nucleus	0.023
	*abpD* (Actin Binding Protein)	Actin-binding protein D	Actin binding protein that is developmentally and cAMP-regulated; associates with intracellular membranes	0.086
	*DDB_G0285767*	DDB0347641	Not yet annotated	0.003
	*DDB_G0346831*	DDB0346834	Not yet annotated	0.014

### Regulatory elements and transcription factor binding motifs common across mammalian HAP1 gene promoters

A computational promoter analysis was performed to identify transcription factor binding sites conserved across mammalian HAP1 gene promoters. The rationale was that the presence of a regulatory element within *HAP1* upstream promoter sequences of multiple species increases its likelihood of being functionally relevant, and knowledge of the type of responses regulated by the corresponding transcription factors may provide insight into the biological pathway(s) in which HAP1 acts. The ‘Common TFs’ function of the Genomatix MatInspector software [50] was used to identify putative transcription factor binding sites within promoter sequences from human, rat, mouse, dog, horse, rabbit, opossum and Tasmanian devil. For human, rat and mouse, sequence consisted of 600 bp upstream and 120 bp downstream of, and including, the single transcription start site (TSS) indicated in the Ensembl genome browser. For dog, horse, rabbit, opossum and Tasmanian devil the TSS was not annotated so 720 bp immediately upstream of the start codon (ATG) was used since in human and rodents the start codon was within the first exon. These regions of sequence were thought likely to incorporate the core promoter region, which Genomatix defines as being within 500 bp upstream of the first TSS and 100 bp downstream of the last TSS.

Sites that were present in all tested eutherian mammals, and zero, 1, or both marsupials, are listed in Table 2 and fell into the main categories of general regulatory elements (TATA box, XCPE1 elements), cell cycle control (MAX, WT1, E2F, E2F6, E2F7, LRRFIP, MAZR), cell differentiation, growth and development (CDX1, CDX2, EGR1, NGFIC (EGR4), GRHL1, ZNF219), endoplasmic reticulum stress response (ATF6) and respiratory function (NRF1). All sequence positions (relative to the start of translation) and match scores are presented in Additional file 3.

**Table 2:** Promoter elements common to HAP1 gene promoters of all tested eutherian mammals, and 0, 1 or both tested marsupials

	Matrix ID	Description	Function
Present in Eutherians (6/6) AND Marsupials (2/2)	O$VTATA.01	Cellular and Viral TATA box elements	Transcription initiation complex
	O$MTATA.01	Vertebrate TATA binding protein factor (muscle)	Transcription initiation complex
	V$CDX1.02	Caudal-type homeobox 1	Differentiation
	V$CDX2.02	Caudal-type homeobox 2	Differentiation
	V$MAX.03	MYC-associated factor X	Cell cycle
	V$WT1.01	Wilms tumour suppressor	Cell cycle
	V$E2F.02	E2F, MYC activator	Cell cycle
Present in Eutherians (6/6) but NOT Marsupials (0/2)	V$XCPE.01	X gene core promoter element	Core promoter element/Enhancer
	V$ZF5.01	Zinc finger/POZ domain transcription factor	Not yet established
	V$ZF5.02	ZF5 POZ domain zinc finger, zinc finger protein 161	Not yet established
	V$E2F.03	E2F, MYC activator	Cell-cycle
	V$NGFIC.01	Nerve Growth Factor-induced Protein C (EGR4)	Growth
Present in Eutherians (6/6) and ONE Marsupial (1/2)	V$ATF6.01	Member of b-zip family, induced by ER damage/stress	ER Stress response
	V$E2F6.01	E2F Transcription factor 6	Cell cycle
	V$E2F7.02	E2F Transcription factor 7	Cell cycle
	V$EGR1.03	Early Growth Response 1	Growth
	V$GRHL1.01	Grainyhead-like	Differentiation/development
	V$LRRFIP1.01	Leucine-rich repeat (in FlII) Interacting Protein	Cell cycle
	V$MAZR.01	MYC-associated zinc finger protein related factor	Cell cycle
	V$NRF1.01	Nuclear Respiratory Factor 1	Mitochondrial energy production
	V$ZNF219.01	Kruppel-like Zinc finger binding protein 219	Differentiation/development

### Existence of a conserved MAX-CDX1/2-TBP promoter model in mammalian HAP1 gene promoters

**Fig. 4:**
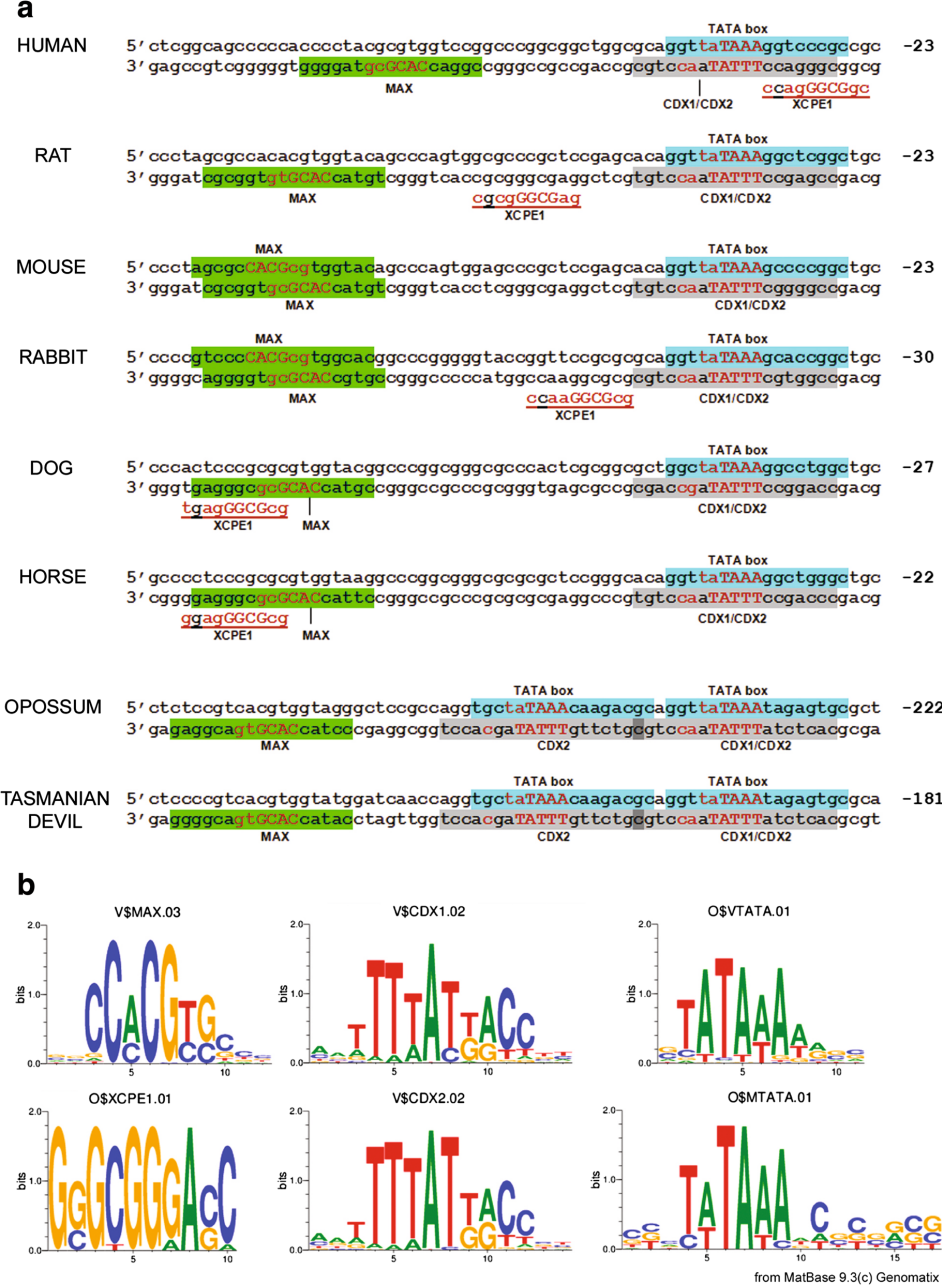
Promoter binding motifs and their location within mammalian HAP1 gene promoters. **a** The core sequence of each binding site is capitalized, and the most informative positions are indicated in red. Positions given are relative to the translation start site (ATG). The TATA box motifs were identified both as ‘Cellular and viral TATA box elements’ (O$VTATA.01) and ‘muscle TATA boxes’ (O$MTATA.01). **b** Sequence logos of the binding site motifs. Total height at each position indicates the information content of that position (in bits), and the relative size of each stacked nucleotide indicates its frequency at that position

The order, position and strand orientation of the transcription factors common to mammalian HAP1 gene promoters were compared between species to identify common patterns. Most notably, there was a site for TATA-binding protein (TBP), also known as the ‘TATA box’, on the sense strand, overlapped on the opposite strand by a site recognized by caudal-type homeobox transcription factors CDX1 and CDX2 (V$CDX1.02, V$CDX2.02). These sites were identically arranged in all HAP1 gene promoters tested here (Fig. 4a). A site for MYC-associated factor X (V$MAX.03) existed upstream, separated by 14–26 bp, on the antisense strand. In mouse and rabbit, this site was overlapped by an additional MAX site on the positive strand, but the site on the negative strand was conserved across all tested mammals. The distance between the conserved promoter model (MAX-CDX1/2-TBP) and the start codon was shorter in eutherian mammals (25–30 bp) than in marsupials (184–207 bp; Fig. 4a). Within the conserved MAX-CDX1/2-TBP model, marsupials contained an additional TATA box upstream of the first, which was absent in eutherian mammals. This upstream TATA box was also overlapped by a CDX2 site (V$CDX2.02) on the antisense strand (Fig. 4a). Five promoter motifs were found only in eutherian HAP1 gene promoters and not in those of marsupials: X gene core promoter element 1 (V$XCPE.01), Zinc finger/POZ domain factor ZF5 sites (V$ZF5.01 and V$ZF5.02), Nerve Growth Factor-Induced Protein C sites (V$NGFIC.01) and E2F sites (V$E2F.03) (Table 2). In 5 of 6 eutherian mammals, an XCPE1 site overlapped the MAX-CDX1/2-TBP model on the antisense DNA strand (Fig. 4a). The consensus DNA sequences for TBP (TATA box), CDX1, CDX2 and XCPE1 are shown as sequence logos in Fig. 4b. A database search for single nucleotide polymorphisms (SNPs) in the human *HAP1* promoter identified *rs116737192*, a SNP that exists at a highly conserved position in the consensus binding motifs of TBP (5′TA[T/C]AAA), CDX1, and CDX2 (5′TTT[A/G]T), such that the minor allele abolishes the prediction of all of these sites by the MatInspector software. The SNP is well represented in the Encyclopedia of DNA Elements (ENCODE) which provided functional supporting evidence from ChIP-seq experiments that TBP, and associated regulatory factors including RNA polymerase II, bind at this genomic location in a number of human cell lines [51]. This SNP is most common amongst individuals of African descent, in which the minor allele frequency is 9.1 % [52].

### Functions associated with human genes with CDX1/2-TBP and MAX-CDX1/2-TBP promoter models

A search of the Genomatix human gene promoter database (112705 sequences) was performed using the ModelInspector program [53], to identify gene promoters containing the CDX1, CDX2 and TBP sites in the identical arrangement as was observed in mammalian HAP1 gene promoters (as described in Materials and Methods). The search resulted in 5956 matches in 5712 sequences (~5 % of human promoter sequences). The most significantly enriched biological functions associated with CDX1/CDX2/TBP genes, represented by Gene Ontology (GO) terms, included processes related to cell-cell adhesion, calcium signaling, cell cycle, ATP catabolic process, purine metabolism, phosphorous metabolism, microtubule-based movement, organelle formation, retinoic acid receptor signaling, and nervous system development (Table 3). Including the upstream MAX site in the model resulted in 68 matches in 67 sequences (~0.06 % of human promoter sequences in the database) corresponding to 55 genes. Enriched biological processes included negative regulation of retinoic acid receptor signaling, negative regulation of apoptosis, cell proliferation, intracellular receptor signaling, development, and detection and transport of calcium (Table 3). Of note, the latter model was found to be common to several PRAME family genes (PRAMEF5, 6, 9, 15, 20 and 25; Table 3) that exist in a cluster of related genes on human Chromosome 1. The commonality of function of these genes is likely to have caused bias amongst the enriched biological processes in this small gene set.

**Table 3:** Overrepresented biological processes associated with human genes with promoter models found in mammalian HAP1 gene promoters

GO-Term ID	GO-Term	Observed # genes	Expected # genes	Fold enrichment	*p*-value	Gene Symbols (MAX-CDX1/2-TBP model only)
Promoter model: CDX1/2-TBP CDX1(neg)-0 bp-CDX2(neg)-2 bp-TBP(pos)						
GO:0007156	Homophilic cell adhesion *via* plasma membrane adhesion molecules	62	32.9	1.9	5.0E-08	
GO:0005513	Detection of calcium ion	12	3.2	3.7	1.2E-06	
GO:0007059	Chromosome segregation	72	46.0	1.6	2.0E-05	
GO:0006200	ATP catabolic process	115	81.9	1.4	3.1E-05	
GO:0007018	Microtubule-based movement	69	44.2	1.6	3.1E-05	
GO:1902115	Regulation of organelle assembly	35	19.1	1.8	7.8E-05	
GO:0048384	Retinoic acid receptor signaling pathway	24	11.5	2.1	9.1E-05	
GO:0019722	Calcium-mediated signaling	45	26.7	1.7	9.4E-05	
GO:1900544	Positive regulation of purine nucleotide metabolic process	170	134.1	1.3	2.7E-04	
GO:0006793	Phosphorus metabolic process	909	831.9	1.1	3.3E-04	
GO:0051301	Cell division	206	167.2	1.2	3.6E-04	
GO:0071840	Cellular component organization or biogenesis	1256	1173.0	1.1	4.8E-04	
GO:0009056	Catabolic process	740	671.2	1.1	5.0E-04	
GO:0022402	Cell cycle process	315	268.4	1.2	5.3E-04	
GO:0007399	Nervous system development	502	446.9	1.1	9.5E-04	
Promoter model: MAX-CDX1/2-TBP MAX(pos/neg)-15 to 60 bp-CDX1(neg)-0 bp-CDX2(neg)-2 bp-TBP(pos)						
GO:0048387	Negative regulation of retinoic acid receptor signaling pathway	6	0.08	75.0	1.0E-10	PRAMEF5, PRAMEF6, PRAMEF9, PRAMEF15, PRAMEF20, PRAMEF25
GO:0043066	Negative regulation of apoptotic process	10	1.97	5.1	1.8E-05	PRDX2, CITED1, KIAA1324, PRELID1, PRAMEF5, PRAMEF6, PRAMEF9, PRAMEF15, PRAMEF20, PRAMEF25
GO:0030522	Intracellular receptor signaling pathway	6	0.71	8.5	6.7E-05	PRAMEF5, PRAMEF6, PRAMEF9, PRAMEF15, PRAMEF20, PRAMEF25
GO:0050793	Regulation of developmental process	14	4.57	3.1	8.2E-05	SHROOM3, HAP1, NEFM, TNFSF9, CITED1, PRELID1, GDF11, FADS1, PRAMEF5, PRAMEF6, PRAMEF9, PRAMEF15, PRAMEF20, PRAMEF25
GO:0008284	Positive regulation of cell proliferation	9	1.91	4.7	9.1E-05	TNFSF9, ADRA2A, TFF2, PRAMEF5, PRAMEF6, PRAMEF9, PRAMEF15, PRAMEF20, PRAMEF25
GO:1901019	Regulation of calcium ion transmembrane transporter activity	3	0.12	25.0	2.4E-04	HAP1, ADRA2A, STIM1
GO:0005513	Detection of calcium ion	2	0.04	50.0	5.6E-04	KCNIP1, STIM1

### Expression of HAP1 in the human duodenum

**Fig. 5:**
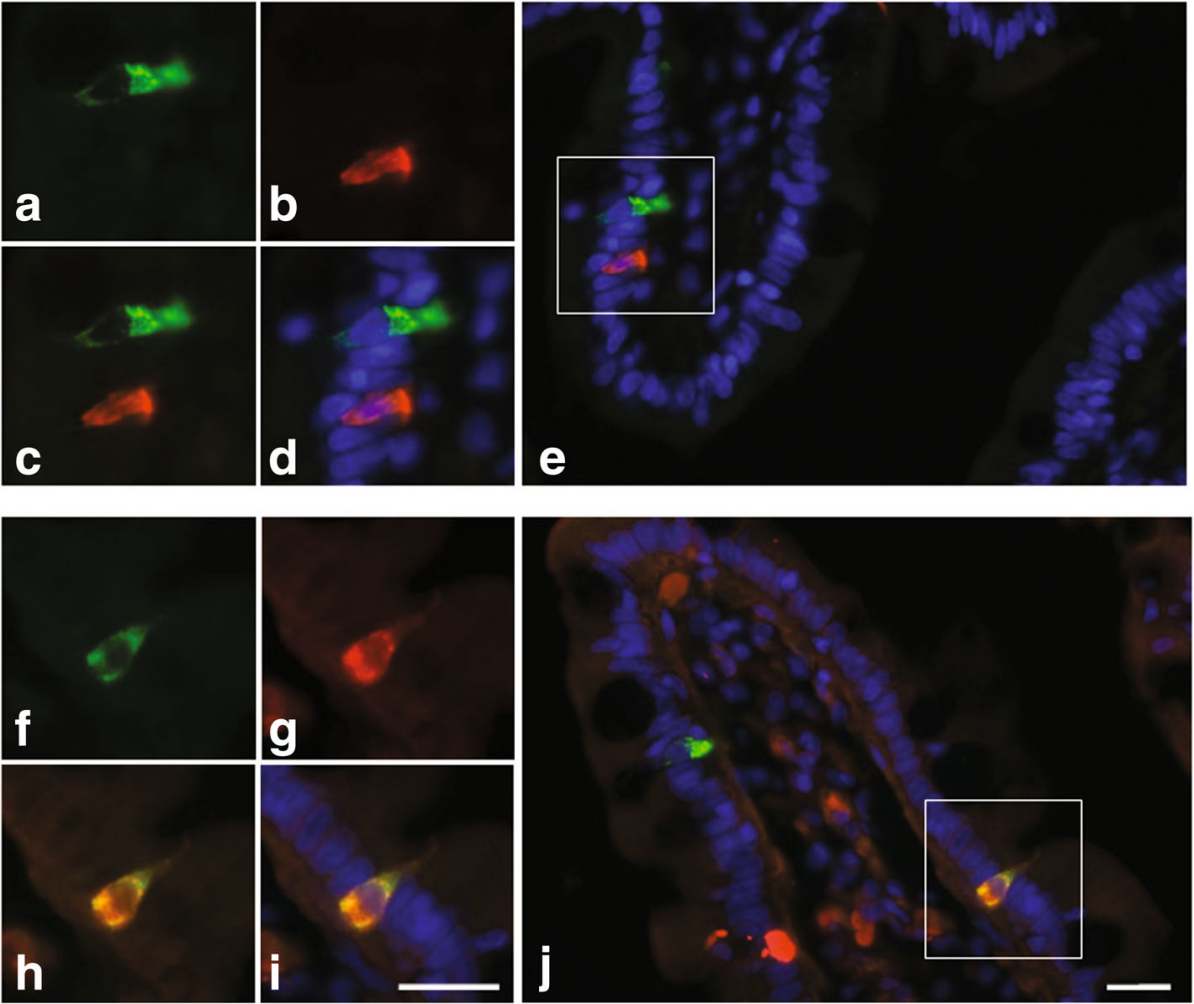
HAP1 and 5-HT localization in villi from healthy human duodenum. (**a-e**) are related images highlighting an example of cells individually positive for HAP1 or 5-HT. Images (**a-d**) are the same field of view (enlarged from **e**), showing HAP1 alone (**a**), 5-HT alone (**b**), merged (**c**), merged with DAPI nuclei stain (**d**), with lumen to the left. Image **e** shows HAP1, 5-HT and DAPI staining in the wider context of the villus. Images (**f-j**) highlight a cell immunopositive for both HAP1 and 5-HT. Lumen is to the top right in (**f-i**). Epifluorescence images taken with 40× magnification. Scale bar = 20 μm

CDX1 and CDX2 are intestine-specific transcription factors differentially involved in enterocyte proliferation and differentiation. The presence of conserved sites for these factors in HAP1 gene promoters prompted us to investigate HAP1 expression in the intestine. HAP1 protein is known to be present in singly dispersed cells of the mucosal layer of the stomach and duodenum of mice [10]. Enteroendocrine cells are dispersed throughout the epithelial lining of the gut wall and it is presumed that the HAP1-positive cells are enteroendocrine cells. However, this has not been confirmed and no information exists on HAP1 expression in the human gut. Using immunolabeling we investigated HAP1 protein expression in the human duodenum. HAP1 was detected in mucosal villi from lean, healthy human subjects, in ~10–20 cells per villus. The highest density labeling was evident at the basolateral membrane (Fig. 5). Due to the abundant number of HAP1-positive cells, we tested whether HAP1 was expressed in the enterochromaffin (EC) cell population. EC cells are the most populous of all enteroendocrine cells and synthesize and secrete 95 % of serotonin (5-hydroxytryptamine, 5-HT) in the body [54]. This cell type is also known to express the transcription factor CDX2 [55]. Co-staining for HAP1 and 5-HT revealed cells singly labeled for either target (Fig. 5, a-e), as well as a subset that were immunopositive for both HAP1 and 5-HT (Fig. 5, f-j). Intracellular distribution of both HAP1 and 5-HT label was punctuate or vesicular, and within co-labeled cells, a degree of overlap in staining was evident (Fig. 5h).

## Discussion

### Insight into HAP1 function from its evolution

HAP1 of rodent and human have been recognized as sharing a region of homology (the HAP1_N domain) with TRAK1 and 2 proteins, and invertebrate proteins Milton (*Drosophila*) and T27A3.1 (*C. elegans*). Previous studies have identified similarities in sequence and function between TRAK1, TRAK2 and Milton which possess an additional domain, the Milt/TRAK domain, further carboxyl to the HAP1_N domain. The *C. elegans* homolog, T27A3.1, has been identified as a homolog of mammalian HAP1 [31]. In this study we compared all the known members of this HAP1_N family, extending this to include additional orthologues from the 5 vertebrate classes.

We have identified (and confirmed by comparison of genomic context), HAP1 orthologues in human, rat, mouse, dog, rabbit, opossum, Tasmanian devil, *Xenopus*, chicken, anole lizard and zebrafish. No additional HAP1_N domain-containing paralogs apart from TRAK1 and TRAK2 were found within these genomes/proteomes. By sequence and domain comparisons we have determined that as recently as the marsupial lineage, HAP1 proteins had a Milt/TRAK domain and were more closely related to paralogs TRAK1 and TRAK2 than to eutherian HAP1.

HAP1_N family members with TRAK domains exist in both vertebrates and *Drosophila*. Within the proteome of the early eukaryote *Dictyostelium*, we found regions of similarity to the HAP1_N domain in actin binding and cytoskeletal proteins but no similarity to the Milt/TRAK domain. We therefore propose that the HAP1_N domain has ancient origins in actin/microtubule attachment and transport functions, and that the Milt/TRAK domain arose post multicellularity but prior to the divergence of protostomes and deuterostomes. Given that HAP1_N proteins with a Milt/TRAK domain (such as Milton, TRAK1 and TRAK2) function in targeted transport of mitochondria to distant cellular regions of energy need, a putative ancestral homolog containing HAP1_N and Milt/TRAK domains may have evolved with the development of longer cell types in multicellular organisms.

In deuterostomes, the number of Milt/TRAK domain-containing HAP1 proteins has increased such that in vertebrates there are three homologs (TRAK1, TRAK2, and HAP1). Since all 5 classes of vertebrate have these 3 paralogs, the increase in paralog number most likely occurred prior to divergence of the vertebrate lineage into these classes. Sequence analyses indicated less similarity amongst the vertebrate Milt/TRAK domain-containing HAP1 orthologues (in zebrafish, anole lizard, chicken, *Xenopus* and opossum), than observed between TRAK1 and TRAK2 orthologues. This suggests HAP1 sequences were already diverging (compared to TRAK proteins) prior to loss of the Milt/TRAK domain in eutherians. However loss of the Milt/TRAK domain may have further altered the selective pressure on the eutherian HAP1 domain, as the latter domain has diverged approximately 3 times more in eutherians than in marsupials, since their last common ancestor.

We found that the Milt/TRAK domain has been lost a second time, in the lineage giving rise to the *C. elegans* homolog T27A3.1. Since only a single HAP1_N-containing homolog has been found in invertebrates such as *C. elegans*, loss of this domain suggests that the function it imparts on HAP1_N domain proteins is not essential for *C. elegans*. Lack of a phenotype when T27A3.1 protein levels are knocked down by RNAi, indicates the T27A3.1 protein itself is not essential for *C. elegans* viability, at least in the absence of stressors [31]. In contrast, the *Drosophila* homolog, Milton, is essential for larval development. It has maintained the Milt/TRAK domain and functions similarly to vertebrate TRAK1 and TRAK2 proteins, in mitochondrial trafficking [32].

To our knowledge, no protein interactions of Milton, TRAK1, or TRAK2 have been specifically attributed to the Milt/TRAK domain, and it remains to be determined how loss of the Milt/TRAK domain, shortening of the carboxyl terminus, and divergence of the HAP1_N domain have altered the function of eutherian HAP1 orthologues. Loss of the Milt/TRAK domain and carboxyl terminus may have affected the ancestral role in mitochondrial trafficking since *Drosophila* Milton interacts with mitochondria *via* sequences within its carboxyl terminal (aa847–1116) [43] and this terminus is lacked by eutherian HAP1 and T27A3.1. Milton also interacts with mitochondria indirectly through binding the mitochondrial protein Miro *via* sequence(s) within the first 750aa, spanning the HAP1_N and Milt/TRAK1 domains [43].

As described earlier, mouse TRAK1, TRAK2 and HAP1 have many common interactors and overlapping functions, largely related to receptor trafficking. However, mouse HAP1 possesses unique and essential functional aspects that are not fulfilled by its paralogs, since ablation of the mouse *Hap1* gene leads to defects in mouse cellular models, and causes postnatal starvation in mice. Mouse HAP1 has pro-catabolic attributes (including supporting autophagosome dynamics [12], inhibiting mTOR signalling [4], and responding to starvation [2]) that are undescribed for TRAK proteins, whilst TRAK1 and TRAK2 have well established roles in mitochondrial trafficking which have not been described for HAP1. Whether these differences can be attributed to the presence or absence of the Milt/TRAK domain and/or carboxyl sequence remains to be explored.

Although not specifically a eutherian phenomenon, mammals are unique amongst vertebrates in that they endure an intense period of starvation between birth and maternal lactation, and upregulate catabolic processes such as autophagy in order to survive this transition [56]. Mice deficient for autophagy gene *Atg5*, present with a similar postnatal phenotype to *Hap1* knockout mice, with no milk visible in their stomachs, and death within 1 day of birth, despite minimal abnormalities at birth [56]. In light of current understanding of HAP1 function, we propose that HAP1 may be important in promoting catabolic processes in periods of severe nutrient deficiency.

### Insight into transcriptional regulation of HAP1 from gene promoter analyses

We utilized computational promoter analyses to gain insight into transcription factors and signaling pathways likely to regulate HAP1 transcriptional activity. We found the analyzed mammalian HAP1 gene promoters to be rich in sites for factors involved in the cell cycle, growth, and differentiation. The predominance of transcription factors involved in cell cycle control (MAX, WT1, E2F, E2F6, E2F7, LRRFIP and MAZR) suggests a key association of HAP1 with this process. HAP1 has been shown to have an inhibitory effect on the growth rate of breast cancer cell lines, and is under-expressed in breast cancer tissues [8]. This negative effect of HAP1 on cell division is consistent with the inhibitory effect of HAP1 on the mTOR pathway that promotes biosynthesis and cell division [4]. HAP1 may also play a direct role in mitosis by facilitating attachment of cargo to microtubules for correct positioning during the cell cycle *via* its interacting proteins that are involved in these processes, including p150^glued^ [35], PCM1 [35, 24] and HTT [57]. Other factors with motifs in the *HAP1* gene promoter have roles in cell differentiation, growth and development (CDX1, CDX2, EGR1, NGFIC, GRHL1, NRF1 and ZNF219). These factors may act upstream of HAP1’s roles in postnatal development [27, 28], neurogenesis, neurotrophin receptor sorting [17], neuronal differentiation [58] and neuritic outgrowth [15, 59]. Binding sites in the mammalian HAP1 gene promoters that may be responsible for transcriptional activation in response to stressors include sites for Activating Transcription Factor 6 (ATF6) and Nuclear Respiratory Factor 1 (NRF1). ATF6 is a key transcriptional activator involved in the endoplasmic reticulum stress response [60]. NRF1 activates transcription of key metabolic genes important for mitochondrial function may act in concert with peroxisome proliferator-activated receptor γ coactivator 1α (PGC1α), a transcription factor responsive to changes in energy balance [61].

TATA box motifs were present in all mammalian HAP1 gene promoters tested. This motif, named after its consensus DNA recognition sequence (5′ TATAAA), is found in approximately one third of vertebrate promoters [62]. It is the binding site for TATA-binding protein (TBP), facilitating binding of the basal RNA polymerase II transcriptional complex for initiation of transcription. In eutherian HAP1 promoters, we identified a TATA box on the sense strand, starting 38 to 46 nucleotides upstream of the open reading frame (ORF), depending on the species. In both human and mouse the TATAAA motif was positioned between −29 and −24 with respect to the transcription start site (annotated by the Ensembl genome browser, data not shown), which is an appropriate distance for a functional TATA box. Marsupial *Hap1* promoters (opossum and Tasmanian devil) contained two TATA box motifs in close proximity, further upstream from the ORF than in eutherians (the closest motif starting 197 and 238 nucleotides upstream of the ORF). Marsupial *Hap1* promoters also lacked XCPE1 sites, that were detected on the reverse strand within the TATA box region of most eutherian promoters tested. XCPE1 sites are general core promoter elements that can enhance transcription activated by other factors (such as NRF1) in *in vitro* promoter assays [63].

Notably, sites for MAX, CDX1 and 2, and TBP were found to form a highly conserved promoter model common to all mammalian promoters tested. The model consisted of a TATA box on the sense strand, a site for CDX1 and CDX2 on the antisense strand overlapping the TATA box, and a MAX site shortly upstream. CDX1 and CDX2 are predominantly intestine-specific transcriptional factors involved in intestinal development, proliferation and differentiation, and are differentially expressed along the intestine and in the crypt/villus axis [64]. Myc-associated factor, MAX is also present in the gut, and its gene expression increases along the crypt-villus axis in association with enterocyte maturation [65]. The phylogenetic conservation of these sites in mammalian HAP1 gene promoters suggests expression of this gene may be regulated by CDX and MAX factors during the development and differentiation of enterocytes.

GO-term enrichment associated with other genes with this promoter model (investigated with and without inclusion of the MAX site) identified biological processes potentially regulated by this combination of factors. In addition to processes in which HAP1 has been implicated, such as microtubule-based movement [23], nervous system development [17], catabolism [12], calcium signalling [66], and inhibition of apoptosis [67], these included processes such as cell division, cell adhesion, purine metabolism and retinoic acid receptor signalling, representing novel processes in which HAP1 may be involved. The presence of functional CDX1 and CDX2 sites in close proximity of the TATA box have been previously described in other genes such as *Sonic hedgehog* (*Shh*) [68], *glucose-6-phosphatase* (*G6PC*) [69], *calbindin-D9K* [70] and *clusterin* [71]. In the case of the *G6PC* gene (which is required for the catabolic processes of gluconeogenesis and glycogenolysis), transcription is activated by CDX1, and this activation is inhibited by CDX2, suggesting the existence of a competitive regulatory mechanism [69].

Interestingly, a human SNP (*rs116737192)* was found to occur at a critical nucleotide in the binding motifs for TBP, CDX1 and CDX2 in the *HAP1* promoter. Individuals homozygous for the minor allele present at expected Mendelian frequency amongst African populations ([72] F. Tekola-Ayele, NIH, personal communication) indicating that the variant does not significantly compromise survival. Given the functional data from ENCODE ChIP-seq experiments indicating TBP (and RNA polymerase II) binds at this position [51], and our finding that the minor allele abolishes the TBP binding motif, this SNP could potentially alter *HAP1* gene expression and act as a modifier of conditions with which HAP1 is associated.

### Insight from localization of HAP1 in the human gut mucosa

Whilst HAP1 protein has been detected in the stomach and duodenum in mouse [10], localization to a specific cell type within the intestine has not previously been confirmed, and the presence of HAP1 in human intestine has not been explored. Here we demonstrated the presence of HAP1 protein (and thus *HAP1* promoter activity) in singly dispersed mucosal cells in the human duodenum. Moreover, a subset of these cells were serotonin (5-HT)-containing EC cells, which also express CDX2 [55]. Expression in EC cells is noteworthy, as not only is gut 5-HT important in regulating gastrointestinal functions such as the modulation of gut motility [73–75], gathering evidence supports a role of gut-derived 5-HT in metabolic regulation and energy balance [76–78]. Furthermore, EC cells are responsive to nutrients including glucose [79, 80], and increase their synthesis of 5-HT during periods of starvation [77], likely through a mechanism that senses reduced glucose availability [80]. Interestingly, we found intracellular co-localization of HAP1 and 5-HT in EC cells occurred in punctate vesicles at the basolateral membrane, suggesting a role for HAP1 in the 5-HT secretory pathway. EC cells release vesicular 5-HT *via* a unique mechanism involving a small fusion pore [81, 82]. Whether HAP1 regulates this process of exocytosis as it does in other endocrine cells [25, 26] is currently unknown.

## Conclusions

In this study we have identified eutherian HAP1 as an evolutionarily recent adaptation of a vertebrate TRAK protein-like ancestor. The function of the Milt/TRAK domain, how loss of this domain has affected HAP1 function since divergence from marsupials, and whether/how this provides a selective advantage in eutherians, particularly in the postnatal period, are questions remaining to be addressed. We have identified conserved regulatory elements amongst mammalian HAP1 gene promoters that provide insight into specific transcription factors and biological processes likely to act upstream of HAP1 gene transcription. Finally, prompted by the presence of a conserved promoter model including sites for intestinally expressed transcription factors (CDX1, CDX2) in the TATA box region of the promoter, we assessed HAP1 expression in the human small intestine. We detected the protein in singly dispersed mucosal cells, including within a subset of serotonin (5-HT) positive EC cells, with partial intracellular colocalization suggesting a role for HAP1 in peripheral 5-HT secretion.

## Materials and methods

### Database searches

Gene information for synteny analysis was acquired from the Ensembl genome browser [83] first from version 82 and 83 and the National Center for Biotechnology Information (NCBI). *HAP1* genes examined were human (*H. sapiens* ENSG00000173805), rat (*R. norvegicus* ENSRNOG00000014819), mouse (*M. musculus* ENSMUSG00000006930), dog (*C. familiaris* ENSCAFG00000015920), horse (*E. caballus* ENSECAG00000010380), rabbit (*O. cuniculus* ENSOCUG00000000605), opossum (*M. domesticus* ENSMODG00000014659), Tasmanian devil (*S. harrisii* ENSSHAG00000005125), zebrafish (*D. rerio* ENSDARG00000074508), chicken (*G. gallus* ENSGALG00000023847), anole lizard (*A. carolinensis* ENSACAG00000017899) and clawed frog (*X. tropicalis* ENSXETG00000012489). Protein sequences and domain predictions were obtained where possible from Ensembl, or otherwise from NCBI if a more recent build was available (opossum, Tasmanian devil, anole lizard). Protein sequence alignment and identity comparisons were conducted using the Clustal Omega (ClustalO) algorithm [84]. Phylogenetic tree construction was performed using the MEGA6 software [85], by first aligning HAP1_N domains using ClustalW, then constructing the tree using the neighbor-joining method (Gonnet matrix). The tree was rooted by assigning the *Drosophila* Milton HAP1_N domain sequence as the outgroup, and clustering outcomes were tested by bootstrapping (1000 replicates). Protein sequence comparisons were performed using the following sequences (coordinates used for the HAP1_N domain phylogeny are indicated in Fig. **3**): human (*H. sapiens* HAP1: ENSP00000334002.4, TRAK1: ENSP00000328998.5, TRAK2: ENSP00000328875.3), opossum (*M. domesticus* HAP1: XP_007482271.1, TRAK1: ENSMODP00000013909.4, TRAK2: ENSMODP00000018999.3), Tasmanian devil (*S. harrisii* HAP1: XP_012403736.1), rat (*R. norvegicus* HAP1: ENSRNOP00000072494.1), dog (*C. familiaris* HAP1: ENSCAFP00000023416.4), zebrafish (*D. rerio* HAP1: ENSDARP00000099219.3, TRAK1: ENSDARP00000115803.1, TRAK2: ENSDARP00000133065.1), clawed frog (*X. tropicalis* HAP1: ENSXETP00000027307.2, TRAK1: XP_012819699.1, TRAK2: ENSXETP00000061179.1), chicken (*G. gallus* HAP1: ENSGALP00000039222.2, TRAK1: ENSGALP00000019447.4, TRAK2: ENSGALP00000013618.4), anole lizard (*A. carolinensis* HAP1: XP_008111605.1, TRAK1: XP_008113765.1, TRAK2: ENSACAP00000001540.3), nematode (*C. elegans* T27A3.1: T27A3.1a.1), fruitfly (*D. melanogaster* Milton: FBpp0297338.1). *Dictyostelium* proteins with regions of similarity to human TRAK1 and *Drosophila* Milton were identified in proteins encoded by the *Dictyostelium* genome [49] by performing BLAST searches [86], *via* dictyBase [87], using a maximum E-value cut-off score of 0.1.

### Identification of *HAP1* promoter elements

Human, rat, mouse, dog and horse promoter sequences were acquired from Ensembl [47]. For opossum and Tasmanian devil, only partial sequence was available from Ensembl, and the start codon was not shown so *Hap1* mRNA sequences from NCBI (XM_007482209.1 and XM_012548282.1, respectively) were used to identify (by BLAST search [86] of genomic builds of the respective genomes) genomic contigs (NC_008802.1 and NW_003838835.1, respectively) from which sequence upstream of the start codon was obtained. The sequence from each species was then entered into the Genomatix software, and the ‘Common TFs’ tool of the Gene Regulation software suite was used to perform searches (of Matrix Library version 9.3) for individual transcription factor matrices (Vertebrate and General Core Promoter elements) common to all entries, using default stringency settings (Minimum Matrix Core similarity, 0.75. Minimum matrix similarity, ‘Optimized’). Match results were extracted and presented with all positions given relative to the translation start codon (Fig. 4a and Additional file 3).

### Overrepresentation of biological processes amongst human genes containing the HAP1 promoter model

Promoter models were defined using the Genomatix FastM [53] tool. The model including CDX1, CDX2 and TBP only, was as follows: V$CDX1.02 (antisense), distance 0 bp, V$CDX2.02 (antisense), distance 2 bp, O$VTBP (sense). The model including MAX, CDX1, CDX2 and TBP was V$MAX.03 (both strands), distance 15–60 bp, V$CDX1.02 (antisense), distance 0 bp, V$CDX2.02 (antisense), distance 2 bp, O$VTBP (sense). Distances between elements represents the number of base pairs between the center of each consensus matrix for the site. The Genomatix ElDorado human gene promoter database (112705 sequences in June 2015 update) was searched for defined promoter models using the ModelInspector software [53], producing a gene list and an analysis of enriched Gene Ontology terms (*p* < 0.01, not adjusted for multiple testing in order to maintain sensitivity [88]) associated with the listed genes. REVIGO software was then used to provide a summarized representative subset of enriched GO terms using a clustering algorithm based on semantic similarity measures [89], and those with a *p*-value less than 0.001 were presented.

### Immunohistochemistry

Duodenal biopsies were collected from 3 healthy subjects, aged 18–75 with no previous gastrointestinal disease and normal renal and kidney function. Subjects fasted overnight then attended the laboratory at 0800. A small diameter video endoscope (GIF-XP160, Olympus, Tokyo, Japan) was passed into the second part of the duodenum, and mucosal biopsies collected using standard biopsy forceps. These were immediately placed into 4 % paraformaldehyde for 2 h, cryoprotected (30 % sucrose in phosphate-buffered saline (PBS)), embedded in cryomolds, and frozen before sectioning at 6–10 μm (Cryocut 1800, Leica Biosystems, Nussloch, Germany) and thaw-mounting onto gelatin-coated slides. HAP1 immunoreactivity was detected using a mouse primary antibody (MA1-46412, 1:1000, ThermoFisher Scientific, Waltham, MA, USA) following antigen retrieval according to manufacturer’s instructions (S1700, Dako Australia, North Sydney, Australia). 5-hydroxy tryptamine (5-HT) was subsequently detected using a goat primary (20079, 1:2000, ImmunoStar, Wisconsin, USA). Immunoreactivity was visualized using species-specific secondary antibodies (1:200 in PBS-Tween 20) conjugated to Alexa Fluor dyes for HAP1 (A11029-AF488, ThermoFisher Scientific) and 5-HT (A11057-AF568, ThermoFisher Scientific). Nuclei were visualized using DAPI mounting media (E36935, ProLong Gold Antifade, Life Technologies, CA, USA).

## Additional files

Additional file 1: Identity matrix showing pairwise percentage amino acid sequence identity between HAP1 family proteins: TRAK1 and TRAK2 (human, opossum, zebrafish, *Xenopus*, anole lizard, chicken), HAP1 (species as above, plus rat, dog, and Tasmanian devil), *Drosophila* (fruitfly) Milton, and *C. elegans* (nematode) T27A3.1. Sequence identifiers are as indicated in Materials and methods. (JPG 1967 kb)
Additional file 2: Sequence alignment of HAP1 family proteins. ClustalO alignment of HAP1 (human, rat, dog), TRAK1 (human, rat, dog), TRAK2 (human, rat, dog), T27A3.1 (*C. elegans*) and Milton (*Drosophila*) proteins is shown. The red boxed region indicates the HAP1_N domain, and the purple boxed region indicates the Milt/TRAK domain. Amino acid color identifies residues that are small/hydrophobic/aromatic (red), acidic (blue) basic (magenta) and hydroxyl/sulfhydryl/amine/glycine (green). Degree of conservation is indicated by an asterisk (*) for complete agreement, a colon (:) for conservation between groups of strongly similar properties, and a dot (.) for conservation between groups of weakly similar properties. Sequence identifiers are as indicated in Materials and methods. (PPTX 3364 kb)
Additional file 3: HAP1 gene promoter element match positions and scores. Excel spreadsheet containing sequence positions (relative to the start ATG codon) and match scores of all conserved promoter elements described in this study. (XLSX 69 kb)

## Abbreviations

5-HT: 5-hydroxytryptamine
CDX1: Caudal-related homeobox factor 1
CDX2: Caudal-related homeobox factor 2
EC: Enterochromaffin
HAP1: Huntingtin-associated protein
HD: Huntington’s disease
HTT: Huntingtin
MAX: Myc-associated factor X
TBP: TATA-binding protein
TRAK1: Trafficking Kinesin protein 1
TRAK2: Trafficking Kinesin protein 2
