# Supplementary figures and images for: Huntingtin-associated protein 1: Eutherian adaptation from a TRAK-like protein, conserved gene promoter elements, and localization in the human intestine
Source: BMC Evol Biol. 2016 Oct 13;16:214. doi: 10.1186/s12862-016-0780-3 (PMC5064798; doi:10.1186/s12862-016-0780-3)

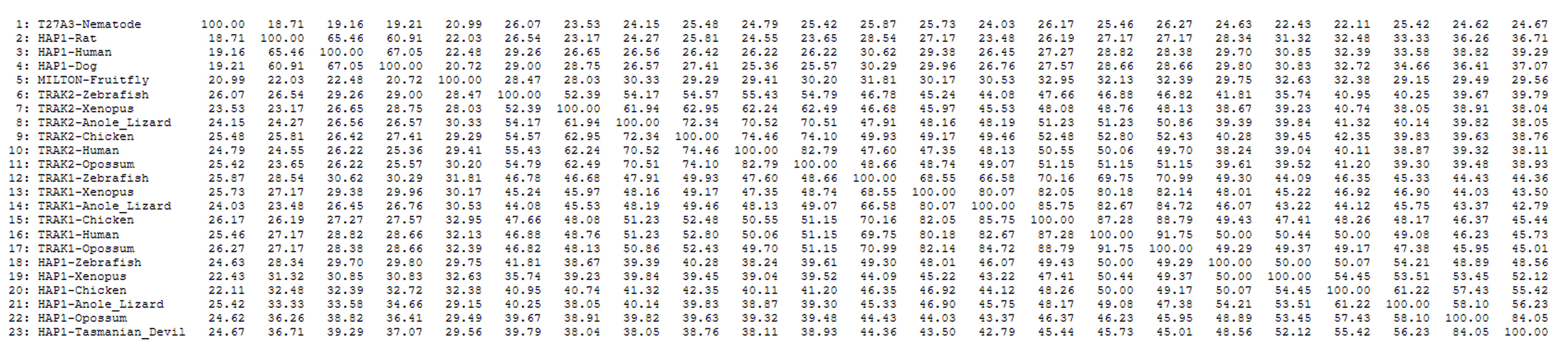

Supplement: Additional file 1: — Identity matrix showing pairwise percentage amino acid sequence identity between HAP1 family proteins: TRAK1 and TRAK2 (human, opossum, zebrafish, Xenopus, anole lizard, chicken), HAP1 (species as above, plus rat, dog, and Tasmanian devil), Drosophila (fruitfly) Milton, and C. elegans (nematode) T27A3.1. Sequence identifiers are as indicated in Materials and methods. (JPG 1967 kb) [file 12862_2016_780_MOESM1_ESM.jpg]
