# Supplementary material for: Huntingtin-associated protein 1: Eutherian adaptation from a TRAK-like protein, conserved gene promoter elements, and localization in the human intestine
Source: BMC Evol Biol. 2016 Oct 13;16:214. doi: 10.1186/s12862-016-0780-3 (PMC5064798; doi:10.1186/s12862-016-0780-3)

## Slide 1
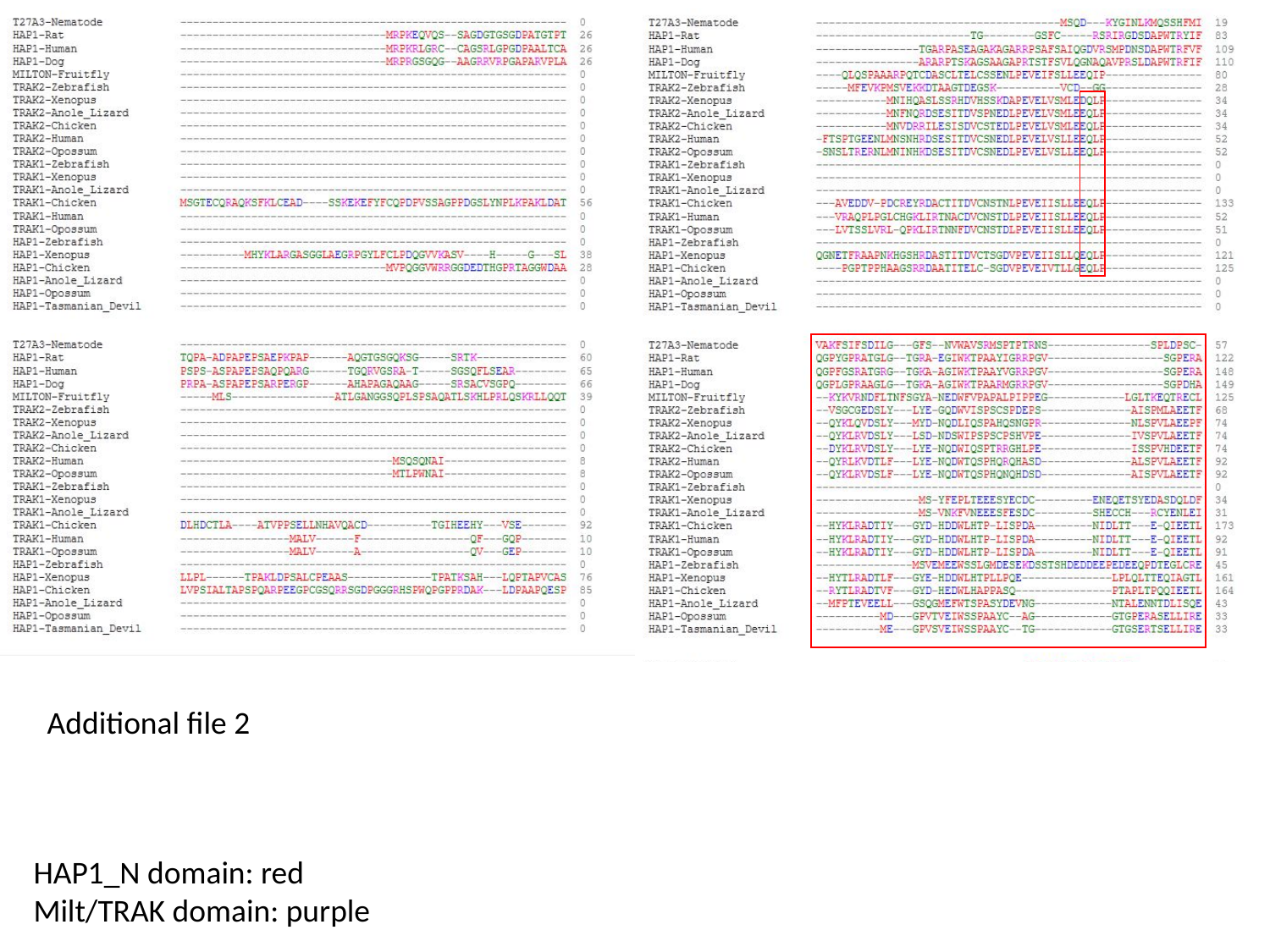

Additional file 2
HAP1_N domain: red
Milt/TRAK domain: purple

## Slide 2
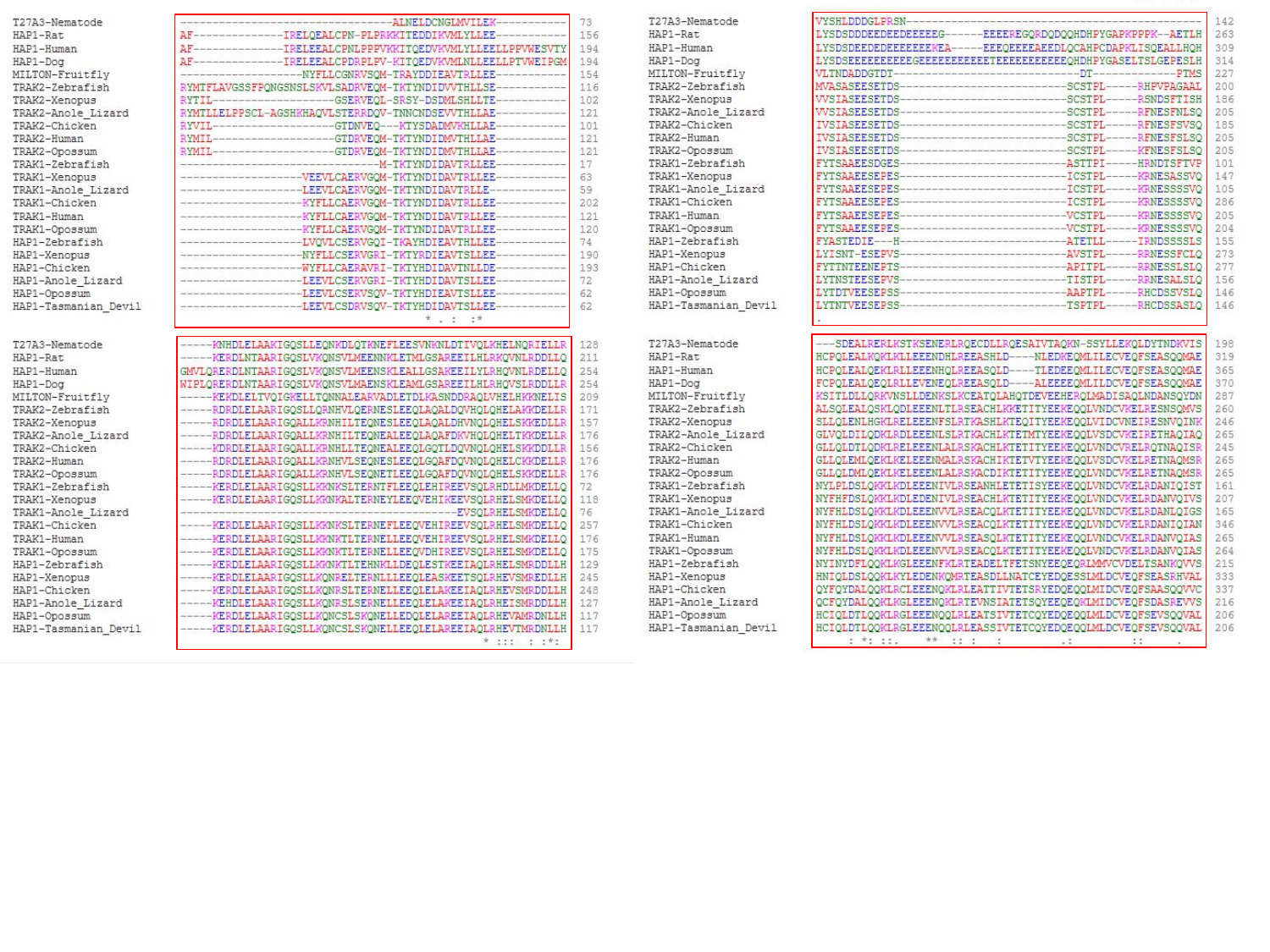

## Slide 3
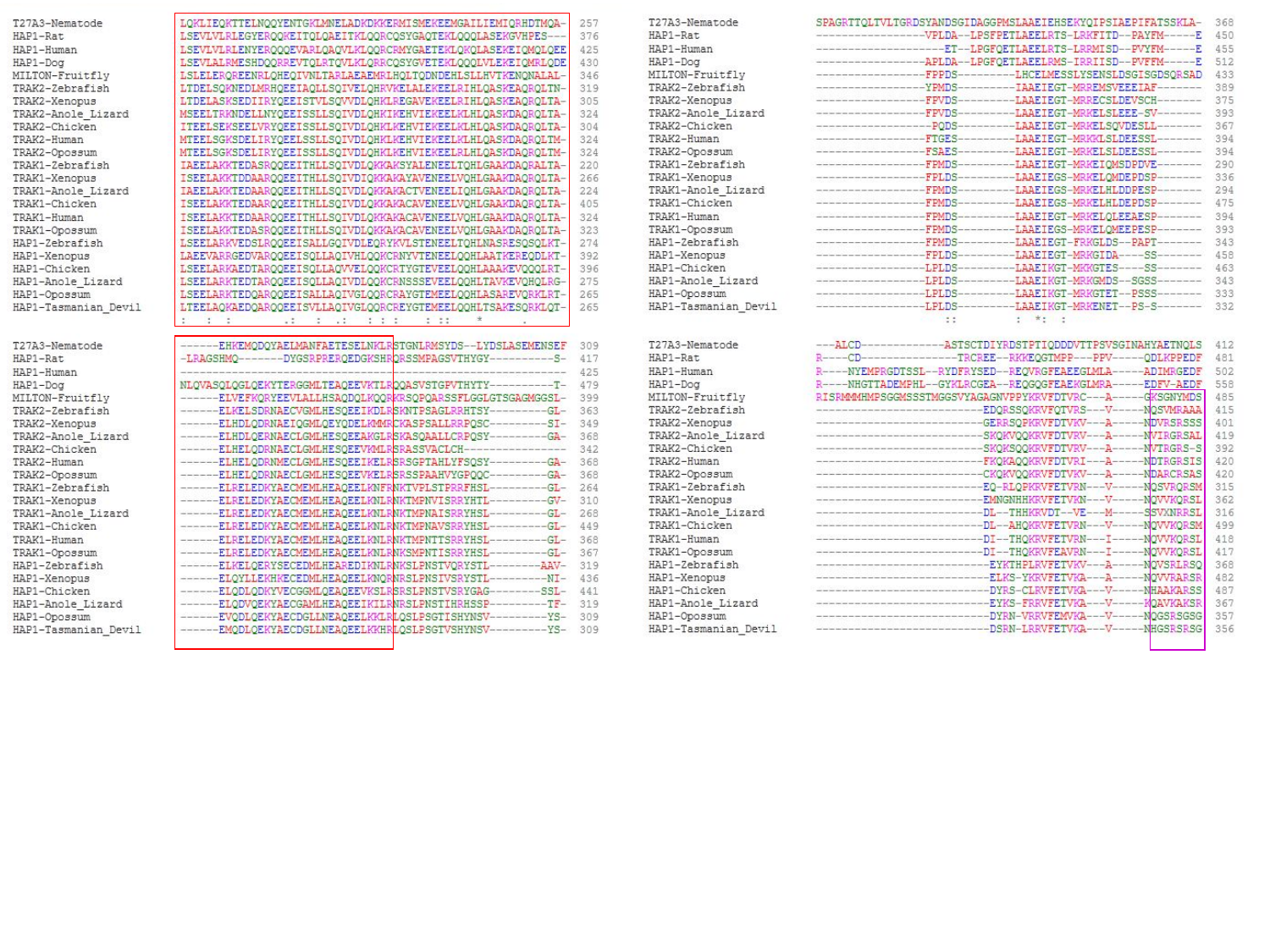

## Slide 4
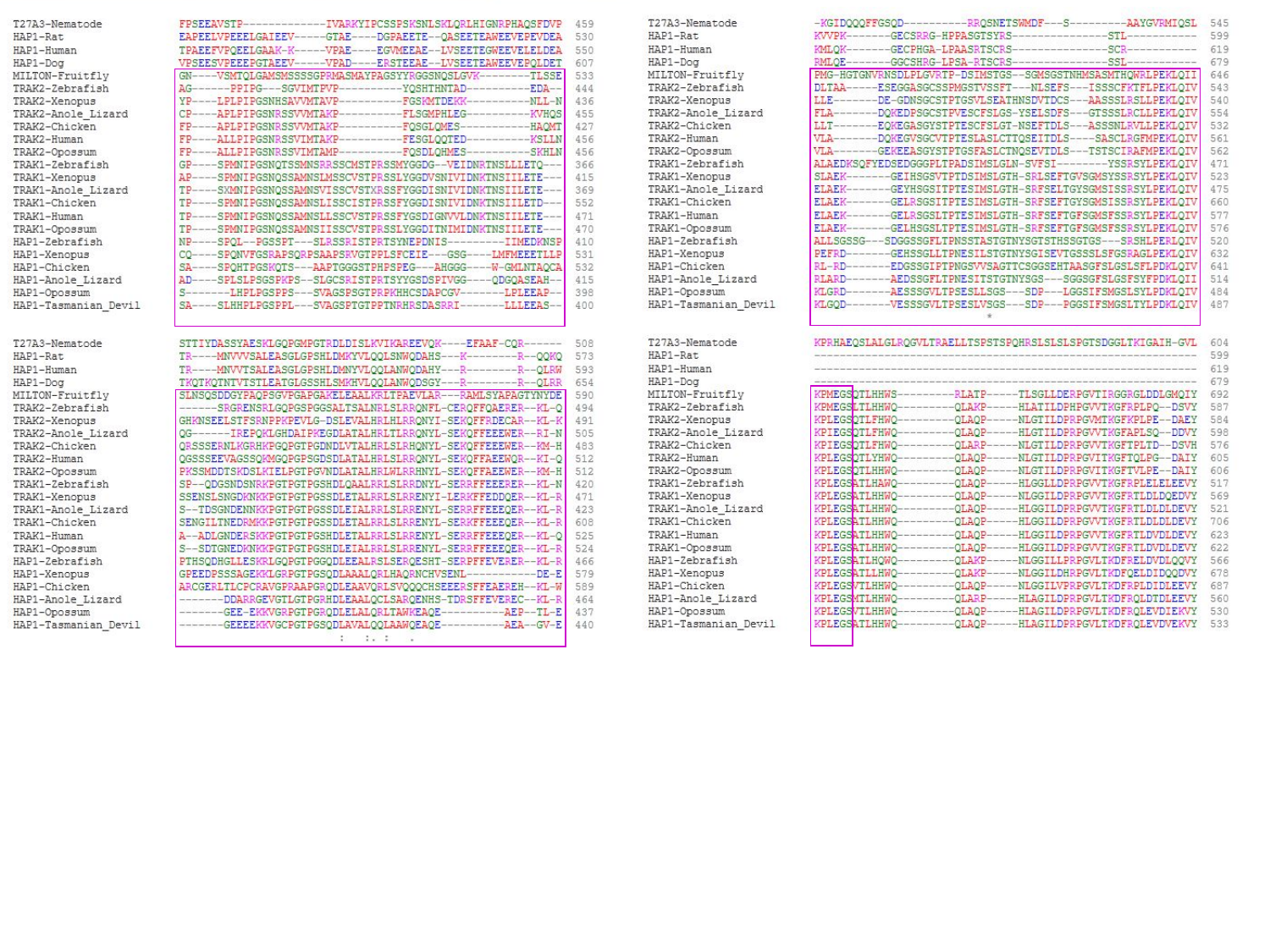

## Slide 5
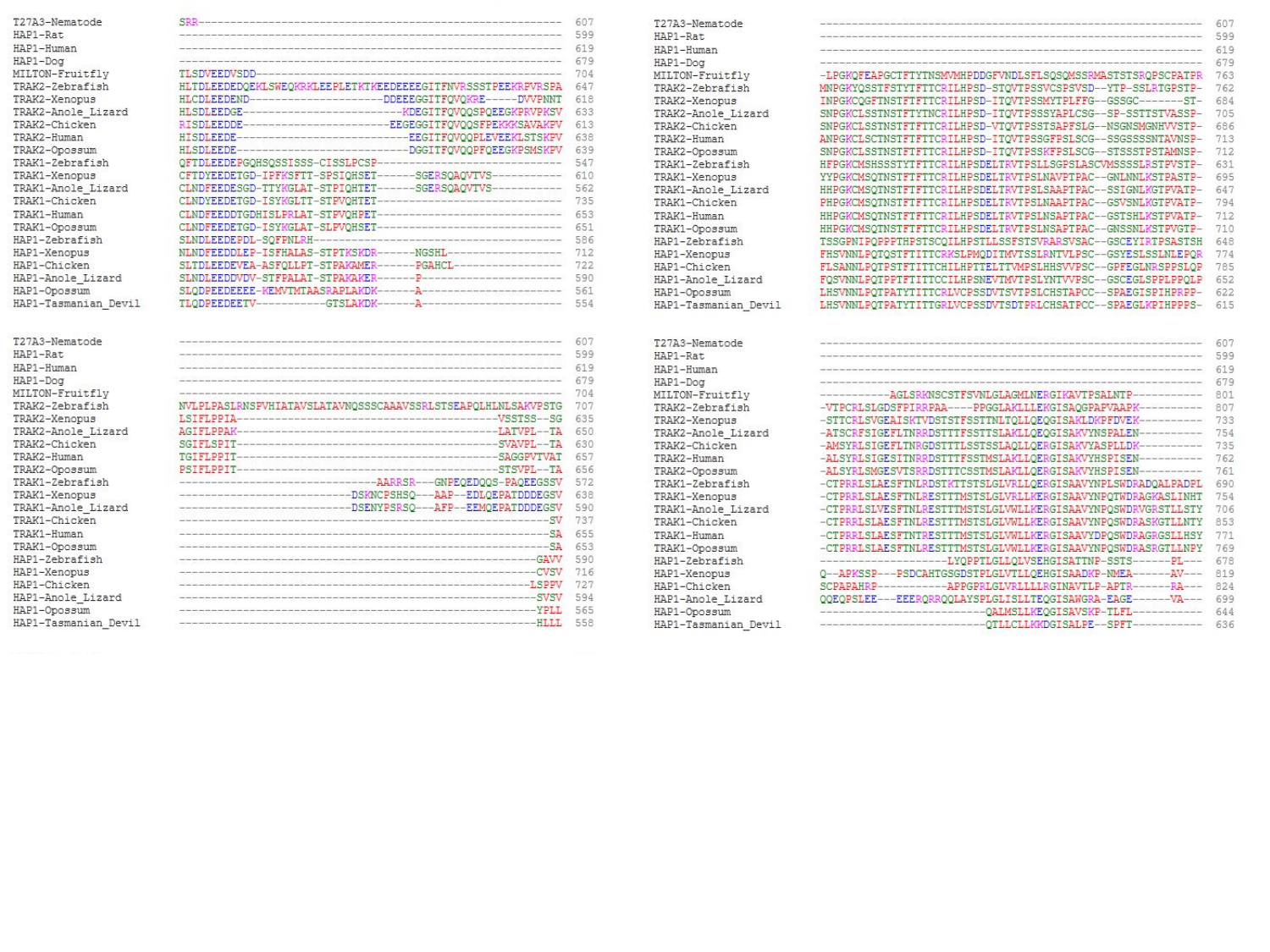

## Slide 6
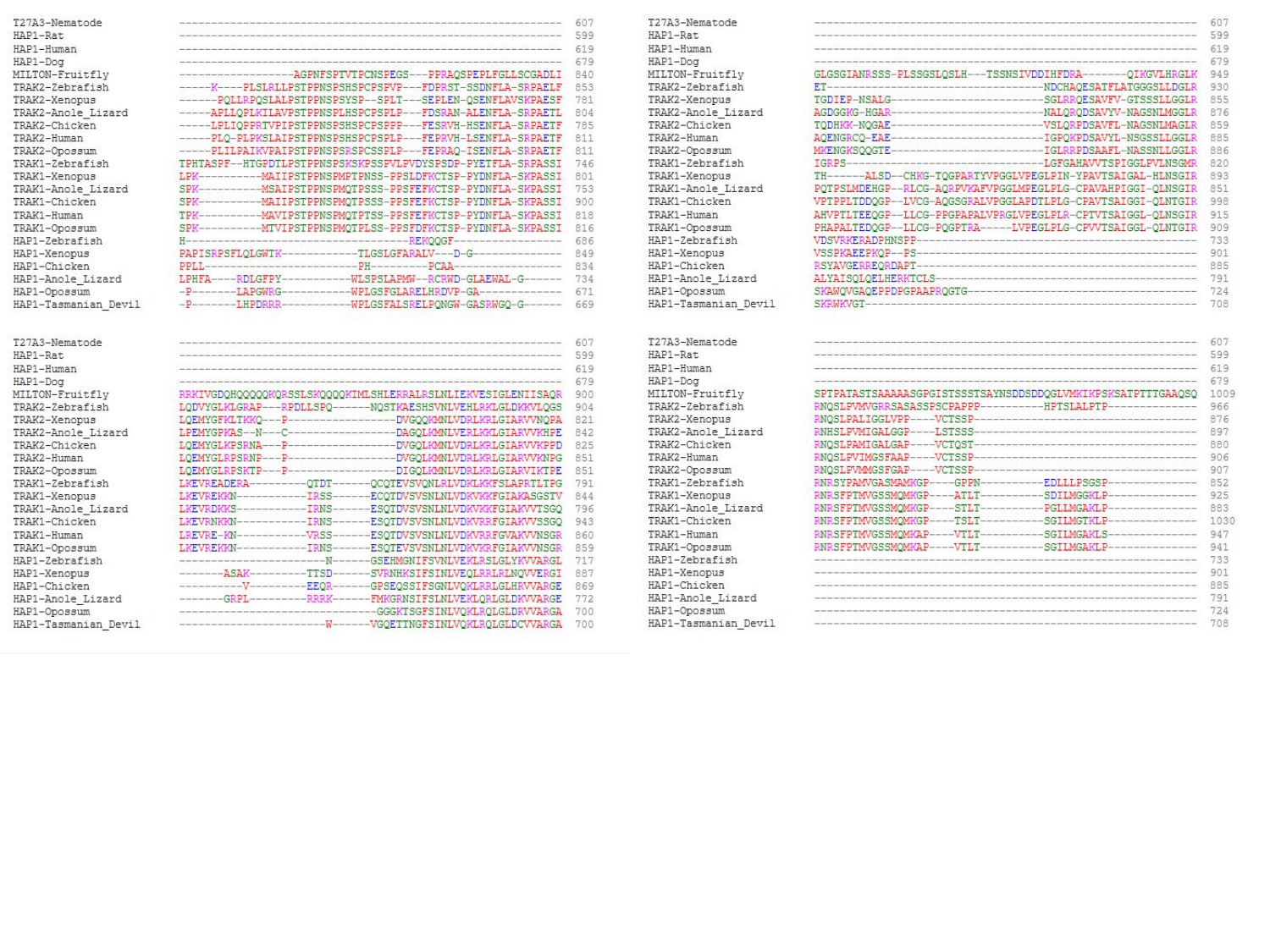

## Slide 7
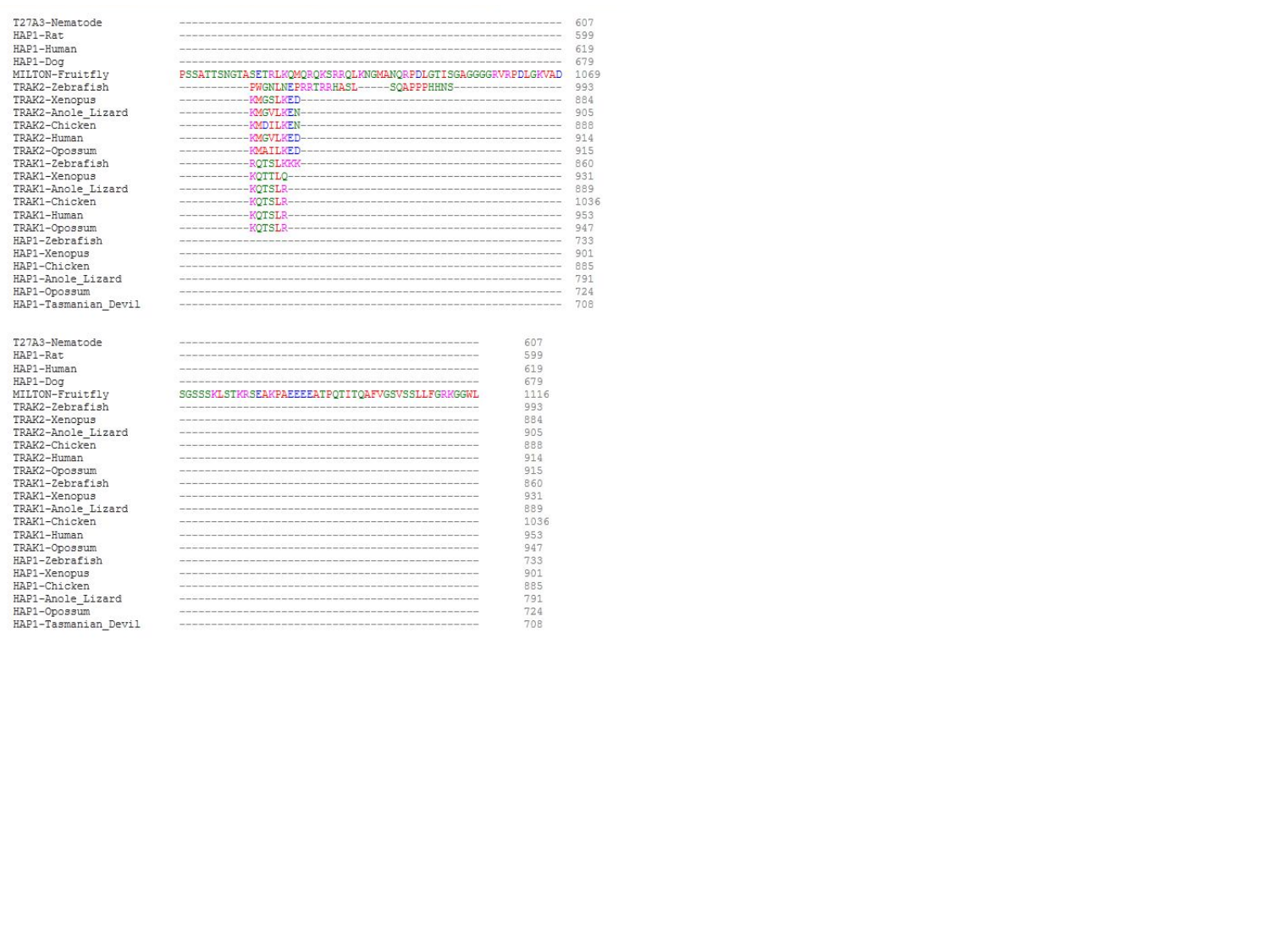

Supplement: Additional file 2: — Sequence alignment of HAP1 family proteins. ClustalO alignment of HAP1 (human, rat, dog), TRAK1 (human, rat, dog), TRAK2 (human, rat, dog), T27A3.1 (C. elegans) and Milton (Drosophila) proteins is shown. The red boxed region indicates the HAP1_N domain, and the purple boxed region indicates the Milt/TRAK domain. Amino acid color identifies residues that are small/hydrophobic/aromatic (red), acidic (blue) basic (magenta) and hydroxyl/sulfhydryl/amine/glycine (green). Degree of conservation is indicated by an asterisk (*) for complete agreement, a colon (:) for conservation between groups of strongly similar properties, and a dot (.) for conservation between groups of weakly similar properties. Sequence identifiers are as indicated in Materials and methods. (PPTX 3364 kb) [file 12862_2016_780_MOESM2_ESM.pptx]
